# Change over time in interactions between unfamiliar toddlers

**DOI:** 10.1177/01650254221121854

**Published:** 2022-09-20

**Authors:** Ayelet Lahat, Michal Perlman, Nina Howe, Holly E. Recchia, William M. Bukowski, Jonathan B. Santo, Zhangjing Luo, Hildy Ross

**Affiliations:** 1 University of Toronto, Canada; 2 Concordia University, Canada; 3 University of Nebraska Omaha, USA; 4 University of Waterloo, Canada

**Keywords:** Toddlerhood, peer relations, longitudinal study

## Abstract

The frequency and length of games, conflicts, and contingency sequences that took place between toddlers as they got to know one another were studied using archival data. The sample consisted of 28 unfamiliar 20- and 30-month-old toddlers (predominantly White, 16 males) who met separately with each of two other toddlers for 18 play dates. The frequency of games increased over time, while the frequency of conflict and contingency sequences decreased. The length of games increased over time while the length of conflicts and contingency sequences were stable. Age and language ability predicted changes in frequency and length of the different types of sequences. Thus, toddlers engage in less structured interactions when they first meet; their interactions become increasingly more organized and positive as the relationship evolves.

Studying the formation of very early peer relationships is important as these initial interactions set the stage for the quality of later peer relationships (Hay et al., 2018; Rubin et al., 2003). Currently, we know very little about the development of peer relationships during the toddler years. Furthermore, it is more challenging to study toddlers who are in the process of forming new relationships than to observe children interacting with familiar peers in child care and other settings (Rubin et al., 2015). This study used a unique archival longitudinal dataset to examine the formation of peer relationships among 20- and 30-month-old toddlers who did not know one another prior to their involvement in this study. Specifically, we examined the frequency and length of different types of interactions between toddlers, and whether these changed over time as toddlers became acquainted with one another. We also examined the role of language and age differences in the frequency and length of these interactions, as toddlers undergo dramatic changes in social and language development during this period. To our knowledge, this is the first study to examine trends based on numerous time points in the development of relationships between toddlers.

## The Formation of Toddler Relationships

Peer relations are important for children’s socialization. When interacting with their chosen playmates, children’s interactions are voluntary, reciprocal, and are of fluctuating duration compared to their interactions with other individuals, such as parents and siblings (Howe & Leach, 2018). Most research on children’s relationships has focused on preschool and school-aged children, and very little empirical work has been devoted to the study of relationships among toddlers (Hay et al., 2018; Rubin et al., 2003). Toddlers engage in active social behaviors and show interest in one another within distinct relationships (Hay et al., 2018). Indeed, children in the first 2 years of life are capable of many forms of peer interaction: they imitate one another (Hanna & Meltzoff, 1993; Howe et al., 2018), share toys (Hay et al., 2021), play games (Goldman & Ross, 1978), cooperate to solve problems (Brownell et al., 2006), engage in physical aggression (Côté et al., 2006; Tremblay et al., 2005), and struggle over toys (Hay et al., 2021; Hay & Ross, 1982). Peer relationships provide children with unique learning opportunities because they require toddlers to move the interaction forward in a way that is not needed when interacting with adult partners (Ross et al., 2010).

Friendships have been identified among toddlers based on children’s responsiveness to one another’s behaviors, physical proximity, and the quality of mutual play (Howes, 1988a, 1988b). Behaviourally, toddler friends are more likely than non-friends to initiate and maintain positive social interactions (Ross & Lollis, 1989); friends also engage in more conflict than non-friends; only when conflicts become especially hostile do friendships end (Howes, 1983; Ross & Conant, 1992). Moreover, keeping and losing friends has important ramifications for toddlers’ interactions with other peers. One study tracked toddlers whose friends graduated to the next age level within child care centers (Howes, 1988a, 1988b). Children whose friends left the group were judged to be less competent with their remaining peers and were less likely to engage in pretend play. Based on these data, Howes argued that stable friendships promote more sophisticated interactions between toddlers because they enable children with limited language abilities to understand their partners’ goals and intentions and to anticipate their friends’ reactions to their own actions.

Most studies of peer friendships are focused on already existing relationships (Rubin et al., 2015). One exception to this is a study examining the formation of relationships among initially unacquainted peers (Gottman, 1983). Children in this study were 3 to 9 years old and were followed for three play sessions. Children who later became friends, according to mothers’ reports, “hit it off” in their first play session. Children who hit it off interacted in a connected manner, exchanged information successfully, managed conflict, and established common ground (Gottman, 1983). In the later sessions, self-disclosure about feelings also became an important predictor of friendship formation. Although these findings do illuminate the formation of relationships between children, the study only explored these relationships over three sessions and did not examine toddlers. The present study will examine the formation of relationships in a younger age group (20 and 30 months) over a longer period of time (18 sessions at each age). It is notable, however, that the behaviors that we study mirror, to some extent, Gottman’s focus on responsiveness, connected discourse, and conflict. It is likely that even among younger participants the interaction will become more organized and include positive exchanges that will increase over time. However, we do not know how long it takes toddlers to get to know one another. Thus, having a large number of sessions used in this study provides an opportunity to track changes in how children interact over a longer period.

Similarly, studies of toddler peer friendships also focused on pre-existing relationships rather than on their development over time starting from when they met for the first time (Hay et al., 2021; Ross et al., 1992). However, two early studies do address changes in toddlers’ peer interaction as relationships form (Hay & Ross, 1982; Muller & Brenner, 1977). In one study (Muller & Brenner, 1977), two playgroups were established in which six toddler boys who met daily for 3 hours over 7 months. Each pair of toddlers within each group was observed on four occasions, spaced evenly over the 7 months. One group of toddlers was slightly older than the other, which allowed the researchers to both compare changes in behavior over the total time period, as well as differences between more or less acquainted toddlers at the same age. The main finding was that coordinated socially directed actions (i.e., sequences in which children responded contingently to the social behavior of the peer) increased linearly over the time that the children spent together. Sequences that involved more than two coordinated actions by the two children also differentiated those who had been meeting together for relatively shorter or longer time periods. In another study (Hay & Ross, 1982), initially unacquainted toddlers were brought together for 15 min play sessions on three consecutive days. On the fourth day, half of the pairs switched partners, whereas half remained with the same partner. This study focused only on children’s conflicts and found no reliable differences in the frequency, duration, or nature of conflict that were attributable to the degree of acquaintance of the two children.

## The Nature of Toddler Peer Interactions: Games, Conflicts, and Contingency Sequences

To be able to engage in sustained interactions with one another, toddlers must draw on multiple emerging social-cognitive skills (Ross et al., 2010). For example, toddlers invite one another to play games that follow organized turn-taking structures and demonstrate their abilities to engage in an activity jointly (Ross, 1982; Tomasello et al., 2005). Playing games (Hay et al., 2018) may be more predictive of relationship formation among older toddlers because children’s developing social-cognitive and language skills increasingly support this type of play. Thus, we will also explore whether the types of interactions, including games, among toddlers forming relationships are moderated by age.

Interactions such as games and conflicts often involve prosocial and aggressive actions. Research on children’s negative behaviors suggests that physical aggression toward peers emerges by the second year of life (e.g., Alink et al., 2008; Baillargeon et al., 2007). Positive behaviors such as cooperating, helping, sharing, and comforting someone in distress begin developing in toddlerhood (Hay et al., 2021). Young toddlers have also been observed engaging in spontaneous helping behaviors (e.g., Warneken & Tomasello, 2006). A recent study (Hay et al., 2021) charted the parallel development of aggression and prosocial behavior from infancy to childhood. The findings suggested a gradual separation of these positive and negative behaviors children engage in when interacting with peers. Furthermore, individual differences in both prosocial behavior and aggression were stable over time.

Games and conflicts have a defined structure; in games this structure is related to roles and repetition, and in conflicts the structure is related to opposition (Goldman & Ross, 1978; Ross, 1982). In games, the roles of the participants are interrelated; roles may imitate the partners’ actions, complement or complete the partners’ actions (e.g., when one child puts a block in a bucket and the other removes the block), or reciprocate the partner’s actions (e.g., when one child throws a ball she has picked up to the partner, and the second picks up and throws it back). In these ways, children’s actions within games are constrained by the roles they adopt, and children take turns that alternate within games and wait while the other child takes a turn. Repetition of roles constrain the actions of the toddlers, but once a game begins these constraints make it easier for the children to continue with the appropriate and repeated actions. Conflicts are constrained by opposition. Especially object conflicts, because there are limits to what one can do to gain access to an object that the peer also wants (Hay & Ross, 1982). Conflict begins with opposition and continues until the opposition has ended (Ross & Conant, 1992).

Alongside games and conflicts, which are organized forms of interactions, other contingency sequences are also building blocks for relationships because they similarly involve responsiveness and continuation of the interaction (Ross & Lollis, 1989). Contingent activity requires that children respond to the socially directed overtures of their peers; they need to understand the meaning of the overture and how to respond to it. Contingency sequences bear some resemblance to categories used in previous work (Gottman, 1983; Muller & Brenner, 1977), which refer to connected interaction and possible successful information exchange, which were important for relationship formation.

Three prior studies have used the archival observational data on which this article is based (Ross, 2013; Ross et al., 1990; Ross & Lollis, 1989). In the first study (Ross & Lollis, 1989), reciprocal relationship effects for contributions to games and contingency sequences were found, while actor and partner effects were present for conflict initiations. That is, initiating a conflict is a characteristic of the individual child, whereas contributions to games and contingency sequences are characteristic of the dyad. Differential relationships emerged gradually over time and were stable and reciprocal only in the latter half of the study. Mothers’ interventions in their children’s property conflicts were examined in the second study (Ross et al., 1990) with findings that mothers’ interventions were frequent and overwhelmingly favored the other child by asking their own child to yield the object to their playmate. The third study (Ross, 2013) was focused children’s behavior in ownership conflicts, demonstrating children’s very early understanding of ownership in that both 20- and 30-month-old owners of objects claimed ownership (“mine”) and instigated and won property conflicts more often than non-owners.

Although this dataset was used in these previous studies, this earlier work did not examine the frequency and length of specific types of behavioral sequences (games, conflict, contingency sequences). The frequency of these sequences provides an estimate of how engaged toddlers are in peer interactions as they become acquainted with one another. Sequence length provides a proxy of toddlers’ ability to sustain a meaningful interaction during a play date. Data in this study will be analyzed using cross-classified multi-level models (CCMMs), which allow us to examine changes over time while considering the variability that can be attributed to session, child, and dyad. Our findings will make a direct contribution to our understanding of the processes that underlie relationship formation in early childhood.

Language skills are important for social development (Longoria et al., 2009; Naerland, 2011). Expressive language skills as manifested in measures such as the mean length of utterance (MLU) predict greater social competence among 18- to 35-month-old toddlers. Furthermore, toddlers with delayed language development showed lower social abilities, compared to age-matched peers (Longobardi et al., 2016). MLU has been proposed to be a better index of expressive language development in children than age (Brown, 1973). MLU is measured by the average number of morphemes made in an utterance. It is calculated by counting the number of morphemes in each utterance divided by the total number of utterances. MLU is used as a benchmark to assess individual differences and developmental changes in grammatical development in children in the early stages of language acquisition.

## The Present Study

Although toddlers can form relationships, little is known about the types of interactions they engage in, or their ability to sustain these different types of interactions. We also do not know how these interactions evolve over time as unfamiliar younger and older toddlers become acquainted. Thus, this study’s objective was to track over time the frequency and length of unfamiliar younger and older toddlers’ interactions.

This study uses an archival dataset to examine the frequency and length of different types of interaction sequences (i.e., games, conflicts, and contingency sequences) between toddlers. We examined whether the frequency and length of different types of sequences varied as a function of age (20 vs 30 months old), and whether these measures changed over time as unfamiliar dyads became acquainted. The frequency indicator provides information regarding the types of interactions toddlers engage in, whereas the sequence length indicator reflects toddlers’ ability to sustain a meaningful interaction, which may be linked to the quality of the relationship as it evolves over time.

Based on past findings that friends engage in more games and conflicts (e.g., Howes, 1983; Ross & Conant, 1992), we expected that the frequency and length of these sequences would increase as children got to know one another. Furthermore, we expected that as toddlers become acquainted over the 18 play sessions, they would be more responsive to one another. Thus, over time, the frequency of contingency sequences would increase, and these sequences would become longer. Given the development of language and social-cognitive abilities that are required for the formation of peer relationships, we expected that older toddlers (i.e., 30-month-old), would have a higher frequency and be able to sustain the different types of interaction sequences for a longer period of time as compared to younger toddlers (i.e., 20-month-old).

## Method

### Participants

The research was approved by the Office of Human Research, University of Waterloo and the Concordia University Office of Research (ethics approval number 30004857). The sample was predominantly White and consisted of 32 pairs of 20- and 30-month-old toddlers (i.e., 16 pairs per age group), who were not familiar with one another at the outset of the study. Pairs of toddlers were arranged into eight groups of four same-age, same-sex pairs. Thus, each child was paired with two others in a same-sex quad (i.e., AB, BC, CD, DA). Age and gender were distributed equally across the quads. Age was a continuous variable, calculated as the toddler’s age in the first session with a peer. At the first session with each peer, toddlers in the 20-month-old quads ranged in age from 18.75 to 24.80 months with an average of 21.24 months; those in the 30-month-old quads ranged in age from 28.35 to 33.95 months with an average age of 30.77 months. The average level of education across the entire sample was 14.18 years for mothers and 16.18 years for fathers, suggesting that most parents in the sample had post-secondary education. None of the children attended organized preschool or daycare. Notably, our sample was homogeneous as none of the children had previous social experiences in group settings, such as toddlers who attended some form of child care. Thus, we were able to examine the formation of new relationships, without the potential confound of other children’s prior experiences with peers in a group setting. Data from a 20-month-old female quad (four pairs), and a 30-month-old male pair were lost since the original data collection and were therefore not available. As such, analyses in this study included data of 28 children formed into 27 dyads.

### Procedure

Data were collected in Waterloo, Ontario in 1983–1984 and several previous studies using the dataset have been published (Ross, 2013; Ross et al., 1990; Ross & Lollis, 1989). Children met in one another’s homes for 18-, 40-min visits, and played together while their mothers were present. Each child had 18 play dates with two other children for a total of 36 play dates. All sessions for a given quad were held within a 4-month period, and sessions alternated between the toddlers’ homes and between visits with the two different partners. Mothers were asked to allow the children to interact freely with one another and not to direct or organize their play; they were free to respond to the toddlers’ overtures. Observations followed procedures established by previous research (Dunn & Munn, 1985).

Five observers worked in various teams of two to collect the data. One pair was responsible for observing all sessions within a dyad with one observer present in the home and recording each play session. The observer followed the children about the home dictating all peer-related social actions onto one track of an audio tape recorder. On a second track, the children’s verbal and vocal behavior was recorded. Live observations with audio records were preferred because of the difficulty of following two children given that they were allowed to move freely throughout their homes. Observers later reviewed the audio tapes and coded the peer-directed actions of each child. In addition, the coded data were accompanied by narrative descriptions of the children’s observed verbal, vocal, and non-verbal actions. Our procedure allowed coding the data for later quantitative analyses and calculation of observer reliability, as well as for others to check the consistency of the coding.

### Coding

The observational sessions were transcribed and coded for peer-directed actions generated by each child. Each action was classified into an interactional sequence between the children that was contiguous in time and thematically related. Preliminary analysis examining the frequencies of sequences indicated that games, conflicts, and contingency sequences had the highest frequency and were, therefore, the focus of this study. Pretend play was examined elsewhere (Luo et al., in press).

Contingency sequences were recorded when children responded to the socially directed actions of their partners and could continue until responsiveness ceased. These sequences included responses that were related to the peer’s prior action. For example, Child A laughed after Child B shot a marble. Another example of a contingency sequence: Child A offered his glass of juice to Child B; Child B laughed; Child A spit and gurgled while drinking juice; Child B did not respond (end of sequence). Games and conflicts also entailed socially contingent responsiveness but were more highly structured, organized, and formed their own two categories of interaction.

Games were defined as structured interchanges in which children have roles to play that are repeated within alternating turns. Game roles could be imitative, complementary (completing an action such as accepting and offering), or reciprocal (as in catching and returning a ball to the partner). During game sequences, toddlers were mutually involved and the sequence had a playful quality without negative affect. For example, Child A started jumping on the couch and Child B joined; both children imitated and followed each other while climbing/jumping on the couch and running around the coffee table. In another example, Child A pushed a car to Child B, who then pushed the car back to Child A, and then the children pushed the car back and forth to each other several more times.

Conflicts were defined by opposition in individual goals and desires, and included protesting, resisting, or retaliating against the actions of another (see the study by Hay & Ross, 1982). Conflict began with opposition and continued until the opposition ended (Ross & Conant, 1992). These sequences included incompatibility of behavior among the toddlers. For example, Child A pushed Child B when he approached; Child B cried and hit Child A. In another conflict example, Child A tugged a wooden car and Child B resisted. Child A then relinquished the car. Each type of sequence included at least two turns—one by each child.

To establish the reliability of the observations, the research coordinator (a sixth observer) was also present and independently recorded 21 (approximately 4%) reliability sessions in total, evenly distributed among the five observers and throughout the data collection period. Reliability was calculated as the proportion of exact agreement (P_A_ = agreement on the occurrence of a sequence/[agreements + disagreements on the occurrence of a sequence]) for the occurrence of each type of coded sequence and exact agreement on the contribution of the children’s actions to a sequence for agreement on sequence length (Syed & Nelson, 2015). P_A_ was preferred to kappa because it is a more conservative estimate of agreement in this case as kappa depends on marginal agreements for the session, including agreements on the non-occurrence of an action, whereas P_A_ is based on agreement on the occurrence of each action within the sequence of ongoing interaction. Therefore, this method does not count as agreements actions that occur at different times and does not depend on agreements on the absence of any one action. P_A_ between observers was .86 for contingency sequences, .87 for games, and .83 for conflicts. Coding disagreements were resolved by consensus between the two observers with reference to the descriptions of the actions transcribed. More general coding decisions were also discussed at weekly coding meetings of the team of observers and supervisors.

### Data Analysis

In this study, two measures were derived for each type of sequence: sequence frequency and sequence length. We were interested in peer interaction, thus actions by mothers within sequences were removed from the analysis. In addition, actions by the same child occurring simultaneously (e.g., a child shouts while she runs away) were combined to create a more accurate measure of sequence length. Sequence frequency was a dyadic measure, as both partners’ interactions were part of a sequence, and was calculated for each session by summing the occurrences of each sequence type. Subsequently, for each child in each session, the number of turns in each game, conflict, and contingency sequence was summed. The sum of the number of turns was divided by sequence frequency within each session, to create an average sequence length measure for each of the 18 sessions. Each child in a dyad contributed a different number of turns to the sequence, and thus sequence length was an individual child measure.

MLU was coded to evaluate each toddler’s verbal ability (Miller & Chapman, 1981). This measure was calculated by using the number of morphemes divided by the number of utterances in toddlers’ interactions. MLU was randomly coded from 3 out of 18 sessions for each toddler (one from each of the first, second, and third thirds of the sessions). The mean of MLU scores from the three play sessions of each toddler was computed as the MLU score for the participant. To calculate MLU reliability, 16 transcripts were randomly selected and coded by a research assistant and one of the co-authors. Reliability was calculated for the number of morphemes and both kappa and percentage agreement were good (0.84% and 92.15%, respectively).

**Figure 1.**
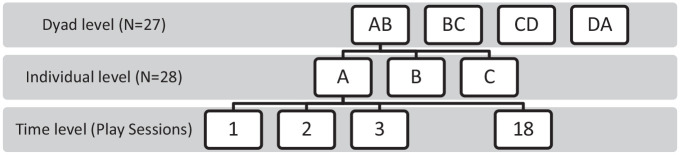
Three-Level Multilevel Model.

Six dependent variables (frequency of games, frequency of conflicts, frequency of contingency sequences, length of games, length of conflicts, and length of contingency sequences) were analyzed using a three-level CCMM to account for the nested structure and cross-classified structure of the data (Leckie, 2013). The CCMM was carried out using Stata/IC Version 16.1. In this study, sessions were nested within child, and children were nested within dyad (See Figure 1). A power analysis was run using Optimal Design Software. The power of finding a statistically significant small effect is 80% based on the sample in this study.

Cross-classified models were used to account for the fact that each child was a member of two different dyads (e.g., Child B is in both dyad AB and dyad BC). The quad level was not included in the model as a level because of its relatively small sample size (*N* = 7). To examine the effect of membership in a quad, separate analyses were carried out, in which each quad was dummy coded and added to the model, respectively, as a predictor. These analyses indicate that some quads were different than others in frequency and length of various sequences. Given the small number of quads, it may not be possible to draw meaningful conclusions from these results (see Supplementary Materials). Frequencies and length of games, conflicts, and contingency sequences for each session of each toddler within each dyad (18 sessions × 2 partners × 27 dyads = 972) were inspected for outliers and identified using Boxplots in SPSS. All outliers (frequencies: 31 games, 12 contingency sequences, and 12 conflicts; length: 20 games, 19 contingency sequences, and 19 conflicts) were replaced by the highest/smallest value in the rest of the data using winsorization (Gwet & Rivest, 1992). Also, the Akaike information criterion (AIC) was calculated to assess model fit because of the small sample size, with an AIC value of 10 considered as meaningful (Vrieze, 2012).

Upon charting each child’s average sequence frequency and length across the 18 sessions for each sequence type, no clear pattern emerged (see Supplementary Materials). It is possible that differences among sessions may be due to noise in the data (e.g., a toddler was tired or hungry in a particular session, toys available in the home, etc.). Therefore, time was aggregated into three relationship phases; the early phase included the first six sessions, the middle phase included the middle six sessions, and the late phase included the final six of the 18 sessions.

Hypotheses regarding change over time were tested by three CCMMs with three levels (i.e., session, child, and dyad). The first model (i.e., the Null Model) did not include any predictors. In Model 1, phase (early, middle, and late) was added as a predictor to investigate the change of interactions over time. The middle and late phase were dummy coded and entered into the model to make comparisons to the early phase. The early and late phase were dummy coded and entered in as a follow-up analysis to compare the early and late phases to the middle phase. These dummy codes allowed us to make all comparisons among the three different phases of the relationship. In Model 2, additional child characteristics were added to the model as predictors and included age, MLU, and interactions between these variables and the different phases.

The level of analysis in this study is the session level nested within individuals within dyads. Thus, the level of analysis includes 972 observations (18 sessions × 2 toddlers in dyad × 27 dyads). However, in Multi-Level Modeling (MLM), the sample size is determined by the number of units in the upper most level in the model. In our case, this is 27 corresponding to the number of dyads in the study. Sample sizes of 30 or larger are considered adequate for MLM. However, this number is not a hard “rule” and while our sample size of 27 is a little lower it is consistent with previous studies that have used similar sample sizes for comparable analyses (Baker et al., 2018; Ram & Grimm, 2009).

A preliminary analysis did not indicate gender differences for any of the sequences, all *p*-values > .12 (see Supplementary Materials for means and *SD*s). Furthermore, no gender differences were found for Child MLU, *p* = .21 (Males *M* = 1.96, *SD* *=* 1.42; Females *M* = 2.58, *SD* = 1.03). Thus, gender was not included in the CCMM. Given that sequence frequency was identical among dyad members and thus dyadic in nature, the average MLU of the dyad was included. Sequence length was slightly different between both members of the dyad because the number of actions was computed at the individual level, and thus each child’s MLU score was entered for this analysis.

## Results

### Descriptive Statistics

Table 1 provides means and standard deviations of sequence frequency and length for each type of sequence by age group. Independent samples *t*-tests indicated that 30-month-old toddlers had significantly more conflicts, *t*(26) = −2.57, *p* = .02, and contingency sequences, *t*(22.36) = −5.58, *p* = .0001 than 20-month-old toddlers, but there were no age group differences for game sequences. Furthermore, conflicts, *t*(24.18) = −2.35, *p* = .03, and contingency sequences, *t*(26) = −4.10, *p* = .0001, were significantly longer for 30-month-old toddlers than 20-month-old toddlers, but there were no significant age group differences in sequence length for game sequences. These findings are partially consistent with our hypothesis that older toddlers would have higher frequency and length for all types of interaction. Our findings were consistent with prior research and Child MLU was higher among 30-month-old toddlers (*M* = 3.11, *SD* = 0.85) compared to 20-month-olds (*M* = 1.05, *SD* = 0.66), *t*(26) = −7.01, *p* = .0001.

**Table 1.** Mean Sequence Frequency and Length (Standard Deviation, Range) by Sequence Type and Age Group.

Sequence type	Sequence frequency			Sequence length		
	20-month-olds (*N* = 12)	30-month-olds (*N* = 16)	95% confidence interval	20-month-olds (*N* = 12)	30-month-olds (*N* = 16)	95% confidence interval
Games	1.62 (0.85, .22–3.31)	1.73 (1.12, .72–4.64)	−.91 to .68	3.76 (1.84, .61–7.28)	4.05 (1.31, 2.01–7.24)	−1.51 to .93
Conflicts	6.87 (2.84, 2.97–11.11)*	9.87 (3.21, 5.14–16.17)*	−5.40 to −.60	3.46 (.41, 2.78–4.0)*	3.98 (.75, 2.94–5.23)*	−.98 to −.06
Contingent actions	5.02 (1.36, 2.25–6.78)**	9.64 (2.92, 5.14–15.22)**	−6.34 to −2.90	2.82 (.73, 1.76–3.86)**	3.99 (.76, 2.94–5.28)**	−1.76 to −.58

*Note.* Age differences across rows.

* *p* < .05; **p < .0001.

### Changes in Sequence Frequency Over Time

#### Contingency Sequences

**Figure 2.**
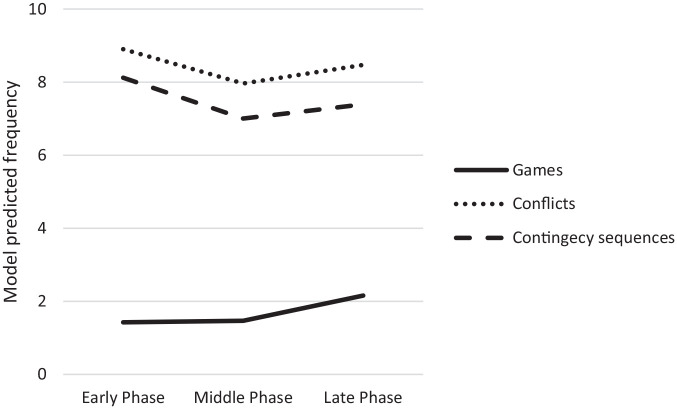
Change Over Time in Frequency of Games, Conflicts, and Contingency Sequences (Model 1). *N* = 28; Values on the Y-axis were calculated by summing the regression coefficient of each phase and the intercept.

When examining the frequency of contingency sequences, significant amounts of the variance were explained at the session and dyad levels in all models. The AIC estimate was lowest for Model 2 (5,289.53) in which 38% of the variance was at the dyad level and 62% at the session level. The proportional reduction in prediction error for the session level from Model 1 to Model 2 was 1.3%. The dummy-coded late phase (*B* = −.73, *p* = .01) and also middle phase (*B* = −1.12, *p* = .0001) both negatively predicted the frequency of contingency sequences. In the follow-up analysis comparing the late phase to the middle phase, the late phase did not predict the frequency of contingency sequences. In contrast to our hypothesis that the frequency of contingency sequences would increase over time, the findings suggest that the frequency of contingency sequences declined from the early to middle phase but did not change from middle to late phase (Figure 2). Also contrary to expectations, there was no effect or interactions with age.

Furthermore, dyad MLU, which was entered in Model 2 significantly positively predicted the frequency of contingency sequences (*B* = 2.24, *p* = .001), such that toddlers with higher dyad MLU scores had more contingency sequences than toddlers with lower dyad MLU scores at the beginning of the relationship. Furthermore, the interactions between dyad MLU and the late phase (*B* = −1.42, *p* = .002) negatively predicted the frequency of contingency sequences, suggesting that participants with lower dyad MLU had a higher frequency of contingency sequences in the late phases of the relationship (Table 2).

**Table 2.** Cross-Classified Multilevel Model Examining Frequency of Contingency Sequences.

	Null model			Model 1			Model 2		
	Estimate	*SE*	95% confidence interval	Estimate	*SE*	95% confidence interval	Estimate	*SE*	95% confidence interval
Fixed effects									
Middle phase				−1.12*	0.27*	−1.66 to −0.58	0.33	2.10	−3.79 to 4.45
Late phase				−0.73*	0.27*	−1.27 to −0.19	−2.80	2.10	−6.92 to 1.32
Age							0.03	0.13	−0.22 to 0.28
Dyad MLU							2.24*	0.65*	0.96–3.53
Age × Middle							0.01	0.11	−0.20 to 0.23
Age × Late							0.19	0.11	−0.02 to 0.41
Dyad MLU × Middle							−0.82	0.46	−1.72 to 0.08
Dyad MLU × Late							−1.42*	0.46*	−2.32 to −0.52
Intercept	7.51*	0.69*	6.15–8.86	8.12*	0.71*	6.73–9.51	2.44	2.61	−2.67 to 7.55
Random effects (variance decomposition)									
Dyad	12.55* (50%)	3.58*	7.18–21.94	12.56* (51%)	3.58*	7.18–21.94	7.34* (38%)	2.22*	4.06–13.27
Child	0.00 (0%)	0.00	0.00–0.00	0.00 (0%)	0.00	0.00–0.00	0.00 (0%)	0.00	0.00–0.00
Session	12.42* (50%)	0.57*	11.35–13.59	12.22* (49%)	0.56*	11.17–13.38	12.06* (62%)	0.56*	11.01–13.20
AIC	5,310.53			5,299.37			5,289.53**		

*Note.* MLU: mean length of utterance; AIC: Akaike information criterion. *N* = 28.

* *p* < .05. **Best model fit based on AIC.

#### Games

When examining the frequency of games, significant amounts of the variance were explained at the session and dyad levels in all models. The AIC estimate was lowest for Model 1 (3,913.15) in which 35% of the random variance was at the dyad level and 65% at the session level. The proportional reduction in prediction error for the session level from the Null Model to Model 1 was 3.6%. In Model 1, the dummy-coded late phase (*B* = 0.73, *p* = .0001), but not middle, positively predicted the frequency of games. In the follow-up analysis comparing the late phase to the middle phase, the late phase (*B* = 0.69, *p* = .0001) positively predicted the frequency of games. These findings suggest that the frequency of games did not change from the early to middle phase, but significantly increased from middle to late phase (Figure 2). Overall, these results are consistent with our hypothesis that the frequency of games would increase over time.

In Model 2, the interaction between age and the late phase (*B* = 0.21, *p* = .0001) positively predicted the frequency of games. These findings are consistent with our hypothesis that older toddlers would have a higher frequency of games than younger toddlers and suggest that this pattern specifically occurs in the late phase of the relationship. Finally, the interactions between dyad MLU and both the middle (*B* = −0.54, *p* = .02) and late phase (*B* = −0.90, *p* = .0001) negatively predicted the frequency of games, suggesting that participants with lower dyad MLU had a higher frequency of games in the middle and late phases of the relationship (Table 3).

**Table 3.** Cross-Classified Multilevel Model Examining Frequency of Game Sequences.

	Null model			Model 1			Model 2		
	Estimate	*SE*	95% confidence interval	Estimate	*SE*	95% confidence interval	Estimate	*SE*	95% confidence interval
Fixed effects									
Middle phase				0.04	0.14	−.23 to 0.31	−1.28	1.04	−3.31 to 0.75
Late phase				0.73*	0.14*	0.47–1.00	−3.02*	1.04*	−5.05 to −.99
Age							−0.06	0.06	−0.17 to 0.07
Dyad MLU							0.17	0.31	−0.44 to 0.76
Age × Middle							0.09	0.05	−0.01 to 0.20
Age × Late							0.21*	0.05*	0.11–0.32
Dyad MLU × Middle							−0.54*	0.23*	−0.98 to −0.10
Dyad MLU × Late							−0.90*	0.23*	−1.34 to −0.45
Intercept	1.68*	0.25*	1.19–2.17	1.43*	0.26*	0.91–1.94	2.52*	1.26*	0.05–4.99
Random effects (variance decomposition)									
Dyad	1.60* (34%)	0.47*	0.90–2.83	1.60* (35%)	0.47*	0.90–2.84	1.56* (35%)	0.47*	0.86–2.83
Child	0.00 (0%)	0.00	0.00–0.00	0.00 (0%)	0.00	0.00–0.00	0.00 (0%)	0.00	0.00–0.00
Session	3.08* (66%)	0.14*	2.82–3.37	2.97* (65%)	0.14*	2.72–3.25	2.93* (65%)	0.14*	2.68–3.21
AIC	3,940.78			3,913.15**			3,923.88		

*Note.* MLU: mean length of utterance; AIC: Akaike information criterion. *N* = 28.

* *p* < .05; **Best model fit based on AIC.

#### Conflicts

When examining the frequency of conflicts, significant amounts of the variance were explained at the session and dyad levels in all models. The AIC estimate was lowest for Model 1 (5,496.78), in which 47% of the variance was at the dyad-level and 53% at the session level. The proportional reduction in prediction error for the session level from the Null Model to Model 1 was 0.80%. In Model 1, the dummy-coded middle phase (*B* = −0.94, *p* = .002), but not late phase, negatively predicted the frequency of conflicts. In the follow-up analysis comparing the late phase to the middle phase, the late phase did not significantly predict the frequency of conflicts. Contrary to our hypothesis that the frequency of conflicts would increase over time, the results suggest that the frequency of conflicts decreased significantly from the early to middle phase and remained stable after that (Figure 2). In addition, contrary to expectations that older toddlers would have a higher frequency of conflicts, there was no effect or interaction with age.

Furthermore, dyad MLU, which was entered in Model 2, significantly positively predicted the frequency of conflicts (*B* = 1.50, *p* = .05), such that toddlers with higher dyad MLU had significantly more conflicts than toddlers with lower dyad MLU at the beginning of the relationship (Table 4).

**Table 4.** Cross-Classified Multilevel Model Examining Frequency of Conflict Sequences.

	Null Model			Model 1			Model 2		
	Estimate	*SE*	95% confidence interval	Estimate	*SE*	95% confidence interval	Estimate	*SE*	95% confidence interval
Fixed effects									
Middle phase				−0.94*	0.30*	−1.54 to −0.34	2.52	2.34	−2.06 to 7.10
Late phase				−0.43	0.30	−1.03 to 0.17	−0.07	2.34	−4.65 to 4.51
Age							0.05	0.14	−0.23 to 0.33
Dyad MLU							1.50*	0.78*	−0.03 to 3.02
Age × Middle							−0.08	0.12	−0.32 to 0.15
Age × Late							0.06	0.12	−0.18 to 0.29
Dyad MLU × Middle							−0.57	0.51	−1.57 to 0.43
Dyad MLU × Late							−0.84	0.51	−1.84 to 0.16
Intercept	8.45*	0.71*	7.04–9.84	8.90*	0.74*	7.46–10.34	4.19	3.01	−1.72 to 10.10
Random effects (variance decomposition)									
Dyad	13.36* (47%)	3.82*	7.63–23.41	13.37* (47%)	3.82*	7.63–23.42	11.70* (44%)	3.44*	6.58–20.82
Child	0.00 (0%)	0.00	0.00–0.00	0.00 (0%)	0.00	0.00–0.00	0.00 (0%)	0.00	0.00–0.00
Session	15.16* (53%)	0.70*	13.85–16.59	15.04* (53%)	0.69*	13.74–16.46	14.89* (56%)	0.69*	13.60–16.30
AIC	5,500.89			5,496.78**			5,499.06		

*Note*. MLU: mean length of utterance; AIC: Akaike information criterion. *N* = 28.

* *p* < .05; **Best model fit based on AIC.

### Changes in Sequence Length Over Time

#### Contingency Sequences

**Figure 3.**
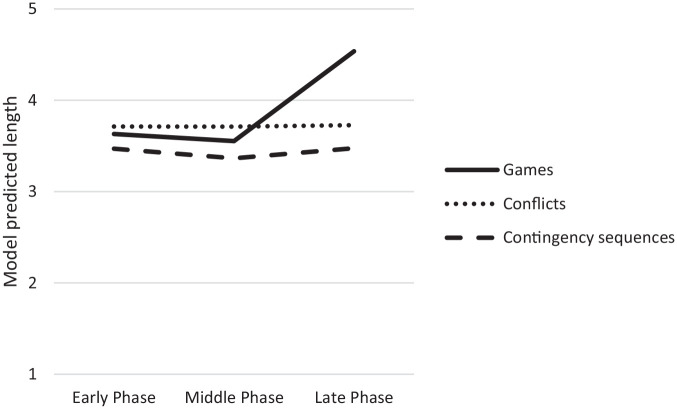
Change Over Time in Length of Games, Conflicts, and Contingency Sequences (Model 1). *N* = 28. Values on the Y-axis were calculated by summing the regression coefficient of each phase and the intercept.

When examining the length of contingency sequences, significant amounts of the variance were explained at the session and dyad levels in all models. The AIC estimate was lowest for the Null Model (3,342.93) in which 35% of the variance was at the dyad level and 65% at the session level. In Model 1, phase of the relationship did not predict changes in length of contingency sequences. Similarly, when comparing the late phase to the middle phase, the late phase did not predict the length of contingency sequences. These results are not consistent with our hypothesis and suggest that length of contingency sequences remained stable over time (Figure 3). Age, which was entered in Model 2 significantly positively predicted the length of contingency sequences (*B* = 0.12, *p* = .0001). Furthermore, the interaction between age and the middle phase negatively predicted the length of contingency sequences (*B* = −0.09, *p* = .006). These findings partly support our hypothesis regarding age and suggest that older toddlers had longer contingency sequences than younger toddlers at the beginning of the relationship. However, older toddlers had shorter contingency sequences in the middle phase than younger toddlers (Table 5).

**Table 5.** Cross-Classified Multilevel Model Examining Length of Contingency Sequences.

	Null model			Model 1			Model 2		
	Estimate	*SE*	95% confidence interval	Estimate	*SE*	95% confidence interval	Estimate	*SE*	95% confidence interval
Fixed effects									
Middle phase				−0.11	0.10	−0.30 to 0.09	2.07*	0.68*	0.74–3.41
Late phase				0.01	0.10	−0.19 to 0.20	−0.42	0.68	−1.76 to 0.92
Age							0.12*	0.03*	0.06–0.19
Child MLU							0.10	0.10	−0.10 to 0.29
Age × Middle							−0.09*	0.03*	−0.16 to −0.03
Age × Late							0.03	0.03	−0.03 to 0.10
Child MLU × Middle							0.11	0.13	−0.14 to 0.35
Child MLU × Late							−0.23	0.13	−0.47 to 0.02
Intercept	3.44*	0.19*	3.07–3.80	3.47*	0.20*	3.08–3.85	0.01	0.77	−1.49 to 1.51
Random effects (variance decomposition)									
Dyad	0.90* (35%)	0.26*	0.51–1.60	0.90* (35%)	0.26*	0.51–1.60	0.45* (22%)	0.14*	0.24–0.83
Child	0.00 (0%)	0.00	0.00–0.00	0.00 (0%)	0.00	0.00–0.00	0.00 (0%)	0.00	0.00–0.00
Session	1.66* (65%)	0.08*	1.52–1.82	1.66* (65%)	0.08*	1.52–1.82	1.64* (78%)	0.08*	1.50–1.79
AIC	3,342.93**			3,351.19			3,351.61		

*Note.* MLU: mean length of utterance; AIC: Akaike information criterion. *N* = 28.

* *p* < .05; **Best model fit based on AIC.

#### Games

When examining the length of games, significant amounts of the variance were explained at the session and dyad levels in all models. The AIC estimate was lowest for Model 1 (5,629.88) in which 16% of the variance was at the dyad level and 84% at the session level. The proportional reduction in prediction error for the session level from the Null Model to Model 1 was 0.88%. The dummy-coded late phase (*B* = 0.91, *p* = .01), but not middle, positively predicted the length of games. In the follow-up analysis comparing the late phase to the middle phase, the late phase (*B* = 0.98, *p* = .01) positively predicted the length of games. These findings are consistent with our hypothesis that the length of games would increase over time, and suggest that although the length of games did not change from the early to middle phase, it significantly increased from the middle to late phase (Figure 3). In Model 2, the interaction between age and the late phase (*B* = 0.23, *p* = .03) positively predicted the length of games. These findings are consistent with our hypothesis regarding age and suggest that older toddlers had longer game sequences later in the relationship (Table 6).

**Table 6.** Cross-Classified Multilevel Model Examining Length of Game Sequences.

	Null Model			Model 1			Model 2		
	Estimate	*SE*	95% confidence interval	Estimate	*SE*	95% confidence interval	Estimate	*SE*	95% confidence interval
Fixed effects									
Middle phase				−0.08	0.33	−0.73 to 0.57	−1.08	2.25	−5.50 to 3.34
Late phase				0.91*	0.33*	0.25–1.56	−4.09	2.25	−8.51 to 0.33
Age							−0.02	0.10	−0.21 to 0.18
Child MLU							0.32	0.33	−0.32 to 0.97
Age × Middle							0.07	0.11	−0.14 to 0.28
Age × Late							0.23*	0.11*	0.02 to 0.45
Child MLU × Middle							−0.41	0.41	−1.22 to 0.41
Child MLU × Late							−0.55	0.41	−1.36 to 0.27
Intercept	3.91*	0.38*	3.16–4.66	3.63*	0.43*	2.79–4.47	3.33	2.34	−1.26 to 7.92
Random effects (variance decomposition)									
Dyad	3.44* (16%)	1.09*	1.85–6.42	3.45* (16%)	1.09*	1.85–6.42	3.42* (16%)	1.11*	1.81–6.45
Child	0.00 (0%)	0.00	0.00–0.00	0.00 (0%)	0.00	0.00–0.00	0.00 (0%)	0.00	0.00–0.00
Session	18.09* (84%)	0.83*	16.53–19.81	17.93* (84%)	0.83*	16.38–19.62	17.88* (84%)	0.83*	16.37–19.58
AIC	5,635.63			5,629.88**			5,645.74		

*Note.* MLU: mean length of utterance; AIC: Akaike information criterion. *N* = 28.

* *p* < .05; **Best model fit based on AIC.

#### Conflicts

When examining the length of conflicts, significant amounts of the variance were explained at the session and dyad levels in all models. The AIC estimate was lowest for the Null Model (3191.22) in which 26% of the variance was at the dyad level and 74% at the session level. In Model 1, phase of the relationship did not predict changes in length of conflict sequences. Similarly, when comparing the late phase to the middle phase, the late phase did not predict the length of conflicts. Contrary to our hypothesis that the length of conflicts would increase as relationships develop, our findings suggest that length of conflicts remained stable over time (Figure 3).

Age, which was entered in Model 2, significantly positively predicted the length of conflicts (*B* = 0.07, *p* = .03). Furthermore, the interaction between age and the middle phase negatively predicted the length of conflicts (*B* = −0.10, *p* = .001). These findings partly support our hypothesis regarding age and suggest that older toddlers had longer conflict sequences than younger toddlers at the beginning of the relationship. However, older toddlers had shorter conflicts in the middle phase than younger toddlers (Table 7).

**Table 7.** Cross-Classified Multilevel Model Examining Length of Conflict Sequences.

	Null Model			Model 1			Model 2		
	Estimate	*SE*	95% confidence interval	Estimate	*SE*	95% confidence interval	Estimate	*SE*	95% confidence interval
Fixed effects									
Middle phase				−0.01	0.09	−0.19 to 0.18	2.47*	0.63*	1.23–3.71
Late phase				0.02	0.09	−0.17 to 0.20	−0.45	0.63	−1.68 to 0.79
Age							0.07*	0.03*	0.01–0.13
Child MLU							0.02	0.09	−0.16 to 0.21
Age × Middle							−0.10*	0.03*	−0.16 to −0.04
Age × Late							0.03	0.03	−0.03 to 0.09
Child MLU × Middle							0.08	0.12	−0.14 to 0.31
Child MLU × Late							−0.17	0.12	−0.39 to 0.06
Intercept	3.72*	0.14*	3.44–4.00	3.71*	0.15*	3.41–4.01	1.86*	0.73*	0.42–3.30
Random effects (variance decomposition)									
Dyad	0.51* (26%)	0.15*	0.28–0.92	0.51* (26%)	0.15*	0.28–0.92	0.45* (24%)	0.14*	0.25–0.82
Child	0.00 (0%)	0.00	0.00–0.00	0.00 (0%)	0.00	0.00–0.00	0.00 (0%)	0.00	0.00–0.00
Session	1.44* (74%)	0.07*	1.31–1.57	1.44* (74%)	0.07*	1.32–1.58	1.40* (76%)	0.06*	1.28–1.54
AIC	3,191.22**			3,201.24			3,206.79		

*Note.* MLU: mean length of utterance; AIC: Akaike information criterion. *N* = 28.

* *p* < .05; **Best model fit based on AIC.

## Discussion

This study examined changes in the frequency and length of interaction sequences among toddlers over 18 play dates. To our knowledge, this is the first study to examine common trends in relationships among toddlers over a prolonged period. We expected that over time, more structured organized interactions would increase and contribute to a reciprocal relationship among toddlers. Furthermore, we expected that older toddlers (i.e., 30-month-olds), would have a higher frequency and be able to sustain the different types of interaction for a longer period of time as compared to younger toddlers (i.e., 20-month-olds).

Overall, our hypothesis that games would become more frequent and longer over time was supported. However, the frequency of conflicts and contingency sequences decreased over the course of the relationship. Furthermore, phase of the relationship was not a significant predictor for change in the length of conflicts and contingency sequences. Thus, the findings suggest that as toddlers become more familiar with each other, more structured and enjoyable activities (i.e., games) increase, while less structured (i.e., contingency sequences) and less enjoyable (i.e., conflicts) activities decrease. As compared to games and conflicts, contingent activity involves a more unstructured action–response pattern, which may decrease when peers are more acquainted with each other and better able to engage in more organized and enjoyable activities. Our findings focus on the process of how toddlers who are initially unacquainted come to structure and put consistent order and regulate their interactions as they become more familiar with their partner.

Thus, different forms of toddler interaction follow different time courses as toddlers form relationships with age-mates. More positive forms of interaction increase over time, as children learn more about the meaning of one another’s positive actions and can then coordinate their own actions with those of a peer. Games increase in length over time as game initiations and signals of intention to play games become recognizable and children learn about the play patterns of others (Goldman & Ross, 1978).

In contrast, conflicts decrease over time, possibly because dominance relationships form, or because children learn to respect the ownership rights of their playmates (Ross et al., 2015; Strayer & Trudel, 1984). Our findings are also consistent with work on developmental trajectories of physical aggression, suggesting that the typical developmental pattern of physical aggression is one of occasional behavior, related to individual and familial differences, which declines over time (Baillargeon et al., 2007; Tremblay et al., 2005). Furthermore, it is possible that changes in conflict and negative behaviors are associated with individual differences that have been found in previous work with similar age groups, such as gender and family background (Baillargeon et al., 2007; Tremblay et al., 2005). Notably, while we measured conflict, physical aggression per se was not measured but was embedded in our definition of conflict. Future research should examine additional child characteristics and individual differences that are linked to conflict among toddlers.

The decrease in contingency sequences and increase in games over time, suggests that as toddlers become acquainted, they are able to engage in interactions that are more organized and require a theme and roles, above and beyond the more literal contingent activity. This finding is consistent with the notion that children’s play ranges from simple physical play actions to more cognitively demanding play, such as imaginative play, which involves more complex scenarios (Howe & Leach, 2018). Our findings indicate that the average frequency of games was overall lower than contingent activity and conflicts, suggesting that toddlers do not yet have the social-cognitive and perspective-taking abilities that allow them to engage in and sustain games to the same extent as conflicts and contingent activity. Our findings are consistent with previous work (Gottman, 1983) and suggest that as the relationship developed, interactions changed from less to more organized. In that early study, self-disclosure, communication clarity, and the exploration of similarity and differences, became increasingly predictive of which children were “hitting it off.”

### Age Differences

Our hypothesis that older toddlers would have higher frequency and length of all types of interactions was only partially supported. We found that older toddlers engaged in more conflicts and contingency sequences than younger toddlers and sustained these two types of sequences longer. We did not find any age-group differences for the frequency and length of game sequences. Consistent with our hypotheses, when age was included as a predictor in the CCMM, the interaction between age and the late phase positively predicted the frequency and length of games, suggesting that older toddlers engaged in more game sequences for longer periods of time later in the relationship. Furthermore, the interaction between age and the middle phase negatively predicted the length of conflicts and contingency sequences, such that older toddlers had shorter conflict and contingency sequences in the middle phase than younger toddlers. The changes over time that were observed in toddler relationships in this study (i.e., increase in complex and enjoyable activities and decrease in simpler and less enjoyable activities) were particularly evident for older toddlers. These changes suggest that less organized, and more negative interactions decreased before positive organized interactions increased.

Age differences in our study may be explained by the children’s development of social-cognition and self-regulation abilities, which support their perspective-taking and help them control actions such as turn taking. A vital component in children’s perspective-taking is the development of Theory of Mind (ToM). ToM refers to children’s ability to attribute mental states to oneself and others and understand that others may have different mental states than their own (Astington & Dack, 2008). The finding that older toddlers engaged in more/longer game sequences later in the relationship is consistent with toddlers’ emerging ability to engage in coordinated interactions (Eckerman & Didow, 1989), and interestingly, our findings add the novel insight that these age-related capacities may in some cases become increasingly evident over time as relationships develop.

Another important developmental process related to young children’s peer relations is emotion regulation. The emergence of emotion regulation is vital to the creation and maintenance of positive relationships with peers (Denham et al., 2003). Children who are skillful in social interactions with peers, particularly those who succeed in negative interactions, effectively regulate their own emotions and subsequent emotion-related behaviors (Denham et al., 2002). This ability to regulate emotions in negative interactions could explain the age differences found in our study.

In addition, the findings that across all sessions, older toddlers were able to sustain conflicts and contingency sequences longer could be explained by their more developed cognitive capacity to focus and sustain attention on one activity for longer periods of time. These findings are consistent with research suggesting increases in sustained focused attention among very young children during play (Ruff & Capozzoli, 2003; Ruff & Lawson, 1990).

### The Role of Language Ability

Age differences in our study could also be attributed to increased language ability among older toddlers. Older toddlers had higher Child MLU scores compared to younger toddlers. Social play requires children to communicate effectively and establish verbal rules that guide their activity (Howe & Leach, 2018). Compared to children with lower communication and language skills, children with increased skills in this domain were better at engaging in prosocial play (Ensor & Hughes, 2005). Furthermore, MLU—a proxy for language competency—has been linked to increased social skills (Longobardi et al., 2016).

Our findings indicated that dyads with higher MLU had a lower frequency of games and contingency sequences later in the relationship. However, MLU was positively associated with contingency sequences at the beginning of the relationship. Although dyad MLU was positively related to conflict frequency at the beginning of the relationship, it did not predict changes over time in conflict sequences. It is possible that dyads with limited verbal abilities can engage in and sustain game sequences, particularly in the later phases of their relationships. That is because games can be non-verbal and, over time, toddlers may become more adept at recognizing overtures to games and sustaining them with age-mates. Similarly, over time, toddler interactions may become more coordinated and the frequency of contingency sequences becomes less dependent on language.

Future research should examine the role of language in the development of young children’s relationship formation. In particular, additional research should be carried out to better understand the role of language in conflicts. It is possible that younger toddlers, with less developed expressive and receptive language, engage in more physical conflict, whereas older toddlers engage more in verbal conflict, as language is more developed among this older age group.

### Limitations and Conclusions

Some potential limitations of this study should be noted. First, although the child/dyad sample size is small, at 28 children, it is close to the 30 units or larger generally recommended for MLM (Baker et al., 2018; Ram & Grimm, 2009). Furthermore, this study included over 1000 play sessions. This intensive longitudinal assessment of each dyad was sufficiently large to produce a very rich and unique set of findings.

Second, other than general demographic information, no other data are available on this sample, with respect to individual differences, such as personality characteristics, child temperament, emotion regulation, physical or mental health. It is possible that these and other individual differences might play a role in relationship formation among toddlers.

Third, because this study used an archival data set, the findings may be subject to cohort effects. Historic changes, such as increased rates of parents joining the workforce have resulted in increased use of formal child care settings. For example, none of the children in this study were attending any formal child care. Thus, the study results may not generalize to children who attend a child care setting and form peer relationships within that context. Yet it should be noted that only 28% of Canadian children aged 1–3 years currently attend formal daycare centers with an additional 13% participating in supervised homecare outside of their own homes (Statistics Canada, 2020). Thus, the experiences of the children in our sample are consistent with those of most Canadian toddlers today. Furthermore, focusing on a sample of children who were cared for at home increased the homogeneity of our sample in terms of their social experiences possibly increasing the likelihood that we could identify trends in relationships as children got to know one another without substantially increasing our sample size. It would be interesting to know if extensive formal child care experience would influence patterns associated with toddlers’ interaction with age-mates.

Finally, parents in our sample were somewhat more educated than the population in Canada. However, it is notable that 86% of Canadians complete high school and 54% of Canadian adults have either a university or college degree (Statistics Canada, 2017).

In summary, this study examined different types of interaction sequences among 20- and 30-month-old toddlers over 18 play dates. The findings indicated that frequency of games increased over time, while the frequency of conflicts and contingency sequences decreased. The length of games increased over time, while the length of conflicts and contingency sequences did not change. Consistent with extant research, age and language ability predicted changes in frequency and length of the different types of sequences. Our findings pertaining to change over time are particularly novel and suggest that toddlers engage in simple forms of interaction when they first meet but that their exchanges become more complex and enjoyable as the relationship evolves over time. Overall, the findings indicate that positive reciprocal relationships are evolving among these toddlers. Supporting children’s repeated opportunities to interact with the same child is important so that they can have these more complex and positive experiences early on in their life. Given the importance of toddler relationships for children’s socialization and later social, emotional, and cognitive development (Hay et al., 2018; Rubin et al., 2006), our findings provide a major contribution to extant literature.

## Supplemental Material

sj-docx-1-jbd-10.1177_01650254221121854 – Supplemental material for Change over time in interactions between unfamiliar toddlers Click here for additional data file. Supplemental material, sj-docx-1-jbd-10.1177_01650254221121854 for Change over time in interactions between unfamiliar toddlers by Ayelet Lahat, Michal Perlman, Nina Howe, Holly E. Recchia, William M. Bukowski, Jonathan B. Santo, Zhangjing Luo and Hildy Ross in International Journal of Behavioral Development
