# Supplementary material for: Change over time in interactions between unfamiliar toddlers
Source: Int J Behav Dev. 2022 Sep 20;47(1):21–34. doi: 10.1177/01650254221121854 (PMC9791325; doi:10.1177/01650254221121854)
Supplement: sj-docx-1-jbd-10.1177_01650254221121854 – Supplemental material for Change over time in interactions between unfamiliar toddlers [file sj-docx-1-jbd-10.1177_01650254221121854.docx]

Supplementary Table 1

Effect of quad on frequency of games

| **Parameters** | Model 3 | Q120_1 | Q130_0 | Q220_0 | Q230_1 | Q330_0 | Q420_1 | Q430_1 |
| --- | --- | --- | --- | --- | --- | --- | --- | --- |
| **Fixed Effects** |  |  |  |  |  |  |  |  |
| Middle phase | .04 | .04 | .04 | .04 | .04 | .04 | .04 | .04 |
| Late phase | .73* | .73* | .73* | .73* | .73* | .73* | .73* | .73* |
| Quad |  | .09 | -.62 | .24 | 1.61* | -.61 | -.54 | -.21 |
| Intercept | 1.43* | 1.41* | 1.52* | 1.39* | 1.19* | 1.51* | 1.51* | 1.45* |
| **Random Effects** |  |  |  |  |  |  |  |  |
| Dyad | 1.60* | 1.67* | 1.62* | 1.66* | 1.31* | 1.62* | 1.63* | 1.66* |
| Child | 0.00 | 0.00 | 0.00 | 0.00 | 0.00 | 0.00 | 0.00 | 0.00 |
| Session | 2.97* | 2.97* | 2.97* | 2.97* | 2.97* | 2.97* | 2.97* | 2.97* |

*Note: N* = 28; **p* < .05

Supplementary Table 2

Effect of quad on frequency of conflicts

| **Parameters** | Model 3 | Q120_1 | Q130_0 | Q220_0 | Q230_1 | Q330_0 | Q420_1 | Q430_1 |
| --- | --- | --- | --- | --- | --- | --- | --- | --- |
| **Fixed Effects** |  |  |  |  |  |  |  |  |
| Middle phase | -.94* | -.94* | -.94* | -.94* | -.94* | -.94* | -.94* | -.94* |
| Late phase | -.43 | -.43 | -.43 | -.43 | -.43 | -.43 | -.43 | -.43 |
| Quad |  | -3.19 | -2.92 | -4.43* | 1.91 | 3.76* | 2.05 | 3.60 |
| Intercept | 8.90* | 9.37* | 9.33* | 9.56* | 8.62* | 8.34* | 8.60* | 8.50* |
| **Random Effects** |  |  |  |  |  |  |  |  |
| Dyad | 13.37* | 12.53* | 12.75* | 11.25* | 13.42* | 12.01* | 13.34* | 12.53* |
| Child | 0.00 | 0.00 | 0.00 | 0.00 | 0.00 | 0.00 | 0.00 | 0.00 |
| Session | 15.04* | 15.04* | 15.04* | 15.04* | 15.04* | 15.04* | 15.04* | 15.04* |

*Note: N* = 28; **p* < .05

Supplementary Table 3

Effect of quad on frequency of contingent actions

| **Parameters** | Model 3 | Q120_1 | Q130_0 | Q220_0 | Q230_1 | Q330_0 | Q420_1 | Q430_1 |
| --- | --- | --- | --- | --- | --- | --- | --- | --- |
| **Fixed Effects** |  |  |  |  |  |  |  |  |
| Middle phase | -1.11* | -1.11* | -1.11* | -1.11* | -1.11* | -1.11* | -1.11* | -1.11* |
| Late phase | -.73* | -.73* | -.73* | -.73* | -.73* | -.73* | -.73* | -.73* |
| Quad |  | -4.43* | -1.82 | -2.35 | 2.91 | 4.32* | -1.98 | 4.27* |
| Intercept | 8.12* | 8.78* | 8.39* | 8.47* | 7.69* | 7.48* | 8.41* | 7.65* |
| **Random Effects** |  |  |  |  |  |  |  |  |
| Dyad | 12.55* | 10.39* | 12.62* | 12.32* | 11.92* | 10.53* | 12.54* | 11.13* |
| Child | 0.00 | 0.00 | 0.00 | 0.00 | 0.00 | 0.00 | 0.00 | 0.00 |
| Session | 12.22* | 12.22* | 12.22* | 12.22* | 12.22* | 12.22* | 12.22* | 12.22* |

*Note: N* = 28; **p* < .05

Supplementary Table 4

Effect of quad on length of games

| **Parameters** | Model 3 | Q120_1 | Q130_0 | Q220_0 | Q230_1 | Q330_0 | Q420_1 | Q430_1 |
| --- | --- | --- | --- | --- | --- | --- | --- | --- |
| **Fixed Effects** |  |  |  |  |  |  |  |  |
| Middle phase | -.08 | -.08 | -.08 | -.08 | -.08 | -.08 | -.08 | -.08 |
| Late phase | .91* | .91* | .91* | .91* | .91* | .91* | .91* | .91* |
| Quad |  | -1.48 | .06 | 1.88 | 1.34 | -.76 | -.92 | -.14 |
| Intercept | 3.63* | 3.85* | 3.62* | 3.35* | 3.43* | 3.74* | 3.77* | 3.65* |
| **Random Effects** |  |  |  |  |  |  |  |  |
| Dyad | 3.45* | 3.31* | 3.61* | 3.12* | 3.36* | 3.53* | 3.49* | 3.60* |
| Child | 0.00 | 0.00 | 0.00 | 0.00 | 0.00 | 0.00 | 0.00 | 0.00 |
| Session | 17.93* | 17.93* | 17.93* | 17.93* | 17.93* | 17.93* | 17.93* | 17.93* |

*Note: N* = 28; **p* < .05

Supplementary Table 5

Effect of quad on length of conflicts

| **Parameters** | Model 3 | Q120_1 | Q130_0 | Q220_0 | Q230_1 | Q330_0 | Q420_1 | Q430_1 |
| --- | --- | --- | --- | --- | --- | --- | --- | --- |
| **Fixed Effects** |  |  |  |  |  |  |  |  |
| Middle phase | -.01 | -.01 | -.01 | -.01 | -.01 | -.01 | -.01 | -.01 |
| Late phase | .02 | .02 | .02 | .02 | .02 | .02 | .02 | .02 |
| Quad |  | -.53 | -.82* | -.58 | .11 | .87* | .19 | .97* |
| Intercept | 3.71* | 3.79* | 3.83* | 3.80* | 3.70* | 3.58* | 3.68* | 3.60* |
| **Random Effects** |  |  |  |  |  |  |  |  |
| Dyad | .51* | .49* | .44* | .49* | .53* | .43* | .53* | .43* |
| Child | 0.00 | 0.00 | 0.00 | 0.00 | 0.00 | 0.00 | 0.00 | 0.00 |
| Session | 1.44* | 1.44* | 1.44* | 1.44* | 1.44* | 1.44* | 1.44* | 1.44* |

*Note: N* = 28; **p* < .05

Supplementary Table 6

Effect of quad on length of contingent actions

| **Parameters** | Model 3 | Q120_1 | Q130_0 | Q220_0 | Q230_1 | Q330_0 | Q420_1 | Q430_1 |
| --- | --- | --- | --- | --- | --- | --- | --- | --- |
| **Fixed Effects** |  |  |  |  |  |  |  |  |
| Middle phase | -.11 | -.11 | -.11 | -.11 | -.11 | -.11 | -.11 | -.11 |
| Late phase | .01 | .01 | .01 | .01 | .01 | .01 | .01 | .01 |
| Quad |  | -1.68* | -.49 | .22 | .45 | 1.21* | -.72 | 1.30* |
| Intercept | 3.47* | 3.72* | 3.54* | 3.44* | 3.40* | 3.29* | 3.58* | 3.33* |
| **Random Effects** |  |  |  |  |  |  |  |  |
| Dyad | .90* | .55* | .91* | .93* | .91* | .74* | .87* | .76* |
| Child | 0.00 | 0.00 | 0.00 | 0.00 | 0.00 | 0.00 | 0.00 | 0.00 |
| Session | 1.66* | 1.66* | 1.66* | 1.66* | 1.66* | 1.66* | 1.66* | 1.66* |

*Note: N* = 28; **p* < .05

Supplementary Table 7

*Mean sequence frequency and length (and Standard Deviation) by sequence type and gender*

| Sequence type | Sequence frequency | | Sequence length | |
| --- | --- | --- | --- | --- |
|  | Females | Males | Females | Males |
| Games | 1.40 (.49) | 1.90 (1.22) | 4.24 (1.39) | 3.69 (1.64) |
| Conflicts | 7.43 (3.59) | 9.45 (3.01) | 3.57 (.76) | 3.90 (.58) |
| Contingent actions | 7.55 (3.03) | 7.74 (3.59) | 3.70 (.74) | 3.32 (1.07) |

Supplementary Figure 1

Average frequency of games over time for each participant

Supplementary Figure 2

Average frequency of conflicts over time for each participant

Supplementary Figure 3

Average frequency of contingency sequences over time for each participant

Supplementary Figure 4

Average length of games over time for each participant

Supplementary Figure 5

Average length of conflicts over time for each participant

Supplementary Figure 6

Average length of contingency sequences over time for each participant
